# Supplementary material for: Spatial distribution and determinants of the change in pre-lacteal feeding practice over time in Ethiopia: A spatial and multivariate decomposition analysis
Source: PLoS One. 2021 Jan 14;16(1):e0244574. doi: 10.1371/journal.pone.0244574 (PMC7808656; doi:10.1371/journal.pone.0244574)
Supplement: Attachment — Submitted filename: Response to reviewers(PFP).docx [file pone.0244574.s001.docx]

Date: December 4, 2020

**Point by point response to editor and reviewer comments**

**Title:** Spatial distribution and determinants of the change in prelacteal feeding practice in Ethiopia; a spatial and multivariate decomposition analysis

**Manuscript number:** PONE-D-20-27150

**Dear editor and Reviewers**: We really thank you for your valuable comments for the betterment of our manuscript. Your concerns and questions as well as suggestions are addressed in the revised manuscript.

**Response to Editorial comment/journal requirement**

1. Please ensure that your manuscript meets PLOS ONE's style requirements, including those for file naming. The PLOS ONE style templates can be found at

**Author’s response:** The author confirm that the revised manuscript meets PLOS ONE's style.

2. We suggest you thoroughly copyedit your manuscript for language usage, spelling, and grammar

**Author’s response:** We extensively edited the manuscript after consulting our colleagues and language experts who had MA degree in TEAFL (teaching English as foreign language) and who had many years’ experience in the area of literature at University of Gondar. A copy of our manuscript showing the changes is indicated by using track changes (See supporting information file).

3. In statistical methods, please clarify whether you corrected for multiple comparisons-

**Author’s response**: Dear editor we consider it in the revised manuscript. In this study, the trends (overall and per each categories of independent variables) were described in the descriptive analysis. As you know in the decomposition analysis, there are two parts, the endowment and the coefficient parts, and we interpreted and discussed the results separately. Moreover, the spatial analysis was conducted using the recent EDHS (EDHS 2016) data.

4. As part of your revision, please complete and submit a copy of the STROBE checklist, a document that aims to improve reporting and reproducibility of observational studies for purposes of post-publication data analysis and reproducibility: (http://www.strobe-statement.org). Please include your completed checklist as a Supporting Information file. Note that if your paper is accepted for publication, this checklist will be published as part of your article.

**Author’s response:** Thank you. We incorporated the STROBE checklist as supporting information (see supporting information).

5. We note that Figures 4, 5, 6 and 7 in your submission contain map images which may be copyrighted. All PLOS content is published under the Creative Commons Attribution License (CC BY 4.0), which means that the manuscript, images, and Supporting Information files will be freely available online, and any third party is permitted to access, download, copy, distribute, and use these materials in any way, even commercially, with proper attribution. For these reasons, we cannot publish previously copyrighted maps or satellite images created using proprietary data, such as Google software (Google Maps, Street View, and Earth).

**Author’s response:** Thank you for the comment. These figures are not copyrighted from other sources rather they are our findings using Arc-GIS version 10.3 and SaTScan version 9.6 statistical softwares. The shape file of Ethiopia was found in the website <https://africaopendata.org/dataset/ethiopia-shapefiles>, and then we generate the figures using the GPs (latitude and longitude) data and the outcome variable using ArcGIS version 10.3 and SaTScan version 9.6 statistical softwares. So all the figures are not copyrighted form other source rather we generate using the software.

**Response to reviewers**

**Reviewer #1:** Dear reviewer thank you in advance for reviewing our paper.

**Reviewer #2:**

1. **Introduction**

The starting paragraphs mostly deal with exclusive breastfeeding and delayed breastfeeding. However, this paper deals with pre-lacteal feeding practices. I would suggest starting introduction with prelacteal feeding and then connect pre-lacteal feeding with delayed breastfeeding and lack of exclusive breastfeeding. The readers would be interested to get to know about pre-lacteal to start of introduction.

**Author’s response:** Dear reviewer thank you for this important concern you raised. We consider you comment and we bother about pre-lacteal feeding in the introduction/background section. We also indicate the relation of pre-lacteal feeding with delayed breastfeeding and lack of exclusive breastfeeding.

**2. Methods**

Method section is well explained. I am just curious that the authors used a number of independent variables i.e. region, residence, perception of distance from the health facility, age, educational level, wealth index, occupation, media exposure, parity, ANC visit, place of delivery, delivery by cesarean section, size of the child at birth, and timing of initiation of breastfeeding and authors ONLY explained two variables in operational definition section.

**Author’s response:** Thank you for the comment. We operationalized media exposure and size of the child at birth to make them measurable and to indicate how these variables were measured to the international readers. However, the rest of the variables are straightforward and there categories are found in the result section (in the tables). Dear reviewer if you are not convinced we are open to consider your comment again.

**3. Result**

The authors presented the results in very details. It is a bit difficult to read and digest the results of all variables. It is also a good idea to just explain your major results and the reader can get the idea of detailed results from the table, and figure and be focused on your major findings. Decrement word is repeatedly used that may be changed to another appropriate word.

**Author’s response:** Thank you for your constructive comment. We put the major findings by avoiding extra and detailed results in the result section of the revised manuscript. In addition, we consider other words for some repeatedly stated words such as “Decrement”.

**4. Discussion**

Discussion section can be improved; for example line 303-306 early initiation of breastfeeding is considered the window of opportunity to decrease the prelacteal feeding. It is important to discuss more and cite some relevant studies to improve this practice. The authors empahsized a lot in the introduction section and they did not put weigh in the discussion section.
Furthermore, different studies report that Delivery by CS is the major determinant of initiation of prelacteal feeding practices in neonate. This study does not report this. It may be important to explain why?

**Author’s response:** Dear reviewer thank you for the important concern you raised. We consider your comment in the revised manuscript. In addition, Cesarean delivery was not associated with the change in pre-lacteal feeding practice in this study (unlike that of the previous studies) and we put a one paragraph statement reveling the discrepancy, the possible reason and further recommendation on this regard (see the discussion section paragraph 10 line 335-341 ).
