# Supplementary material for: Spatial distribution and determinants of the change in pre-lacteal feeding practice over time in Ethiopia: A spatial and multivariate decomposition analysis
Source: PLoS One. 2021 Jan 14;16(1):e0244574. doi: 10.1371/journal.pone.0244574 (PMC7808656; doi:10.1371/journal.pone.0244574)
Supplement: Attachment — Submitted filename: Response to reviewer(#2).docx [file pone.0244574.s002.docx]

**Date: December 11, 2020**

**Response to editor comment**

**Title:** Spatial distribution and determinants of the change in pre-lacteal feeding practice over time in Ethiopia: a spatial and multivariate decomposition analysis

**Manuscript number:** PONE-D-20-27150

**Dear editor:** We really thank you for your valuable comments for the betterment of our manuscript. Your concerns and questions as well as suggestions are addressed in the revised manuscript.

**Point by point response to editor comment**

1. In the Methods section, where operational definitions are presented, it would be prudent to add a sentence similar to that included in the response to reviewer comments, noting "Other independent variable definitions are self-explanatory" and also citing standard DHS survey modules for further reference.

**Author’s response:** We added the sentence and we put the survey module as a reference (See line 120 and 121 of the revised manuscript).

2. Figures 1 and 3 are superfluous (though data on regional distributions is relevant and important). Please consider omitting these figures and integrating regional distribution data as a row in Tables 1 and 2 respectively.

**Author’s response:** Thank you. We consider the comment (see the revised manuscript).
